# Methods for conducting a living evidence profile on mpox: An evidence map of the literature

**DOI:** 10.1002/cesm.12044

**Published:** 2024-02-22

**Authors:** Kusala Pussegoda, Tricia Corrin, Austyn Baumeister, Dima Ayache, Lisa Waddell

**Affiliations:** ^1^ Public Health Risk Sciences Division, National Microbiology Laboratory Public Health Agency of Canada Guelph Ontario Canada

**Keywords:** evidence synthesis, literature surveillance, living evidence, living evidence profile, monkeypox, mpox

## Abstract

**Background:**

In May of 2022, several cases of mpox were identified in multiple nonendemic countries and on July 23, 2022 the World Health Organization declared mpox a Public Health Emergency of International Concern. During the first six months of the outbreak there was an urgent need to have up‐to‐date synthesized evidence on mpox to inform public health decision‐making. At this point, evidence is changing too quickly for traditional evidence synthesis methods, as systematic reviews were out‐of‐date before publication. This paper describes the framework developed to manage and maintain a living evidence profile (LEP) to systematically identify, classify and synthesize evidence on a broad range of mpox topics at a rapid pace as the outbreak unfolded.

**Methods:**

The LEP framework was based on principles of evidence synthesis, risk assessment, priority epidemiological parameters for infectious disease modeling and consultation with experts. The framework consisted of a systematic search conducted twice weekly; study selection; categorization into pre‐determined foci and data extraction; integration and synthesis of evidence; internal peer‐review and dissemination to stakeholders.

**Results:**

Between April 14 and December 15, 2022, 2287 citations were identified, 687 were primary research studies or surveillance reports on the 2022 mpox outbreak and 496 were included in the final LEP. Each study was mapped to one of 32 foci and evidence was narratively synthesized. From June to December 2022, 23 LEPs were produced (approximately weekly) along with a searchable database of extracted data of the mpox literature. They were disseminated globally to public health researchers and decision‐makers to inform public health response efforts.

**Conclusions:**

The LEP framework is applicable to other public health emergencies when a rapid synthesis cycle is required because the evidence is evolving quickly. This efficient methodology for creating up‐to‐date summaries of the current evidence during the first few months of an outbreak or emergency supports public health decision‐making and response activities.

## 1 INTRODUCTION

In May 2022, there was a surge in mpox (formerly known as monkeypox) cases in multiple nonendemic countries with sustained community transmission that were not linked to travel [1]. On July 23, 2022 the World Health Organization (WHO) declared the multi‐country outbreaks of mpox a Public Health Emergency of International Concern (PHEIC) [2] and as of January 1, 2024 there have been 92,783 confirmed cases and 171 deaths reported to the WHO from 116 member states [2, 3]. Since the global weekly case peak of 7576 the week of August 8, 2022 there has been a steady decline in weekly cases reported [2].

Mpox is a zoonotic disease caused by an orthopoxvirus. Infection can result in mild to severe disease in humans and typically includes a pox rash, lymphadenopathy, fever, malaise, and other symptoms that may lead to a variety of complications [2, 4]. Mpox was discovered in the Democratic Republic of Congo in 1970 and before 2022 had been contained to central and western Africa with occasional cases exported to non‐endemic countries and most outbreaks were linked to spillover events associated with infected animal contact [1, 5, 6]. In 2017, a large outbreak in Nigeria resulted in exportation of cases to non‐endemic countries that were linked to human‐to‐human transmission [1, 6]. The mpox virus has been mainly transmitted by direct close contact with skin lesions, body fluids or respiratory droplets, but can also potentially be transmitted via contaminated materials such as bedding or clothes [2, 4, 7, 8, 9, 10].

During the early phase of an emergent public health outbreak (e.g., COVID‐19 and mpox), evidence is rapidly evolving and response activities are constantly being re‐evaluated by public health decision‐makers. During this time, there is a need for timely and continuously updated summaries of all emerging evidence to inform research and response activities [11]. During times of rapid increase in research, conventional evidence syntheses such as systematic reviews and scoping reviews are outdated at publication for decision‐making as they take time to design, produce, and publish and have too narrow a scope or insufficient depth, respectively [11, 12, 13, 14]. Without up‐to‐date evidence syntheses covering a broad range of topic areas, decision‐makers are left to interpret emerging studies without the context of the body of evidence as a whole, impacting trust in policy decisions [11].

The living evidence profile (LEP) was designed to address the challenges of timeliness, depth, and breadth of topic coverage faced by conventional evidence synthesis products while maintaining a structured, reproducible rapid cycle of identifying, extracting, and synthesizing new evidence in the context of what is already known. This paper describes the framework developed to manage and maintain a LEP on mpox during the first six months of the outbreak.

## 2 METHODS

A LEP was initiated on May 27, 2022 after evaluating the need for up‐to‐date evidence by stakeholders within the Public Health Agency of Canada, herein referred to as Agency. The LEP framework was developed by the Agency and utilized structured and reproducible knowledge synthesis methodologies [15, 16]. The content and organization of the evidence in the LEP was informed by hazard profiles from food safety risk assessment, epidemiological parameters needed for infectious disease modeling, consultation with public health experts and knowledge of the important types and categories of studies that were likely to be published given the team's experience with infectious diseases and emerging evidence during the COVID‐19 pandemic [17, 18, 19].

**Figure 1:**
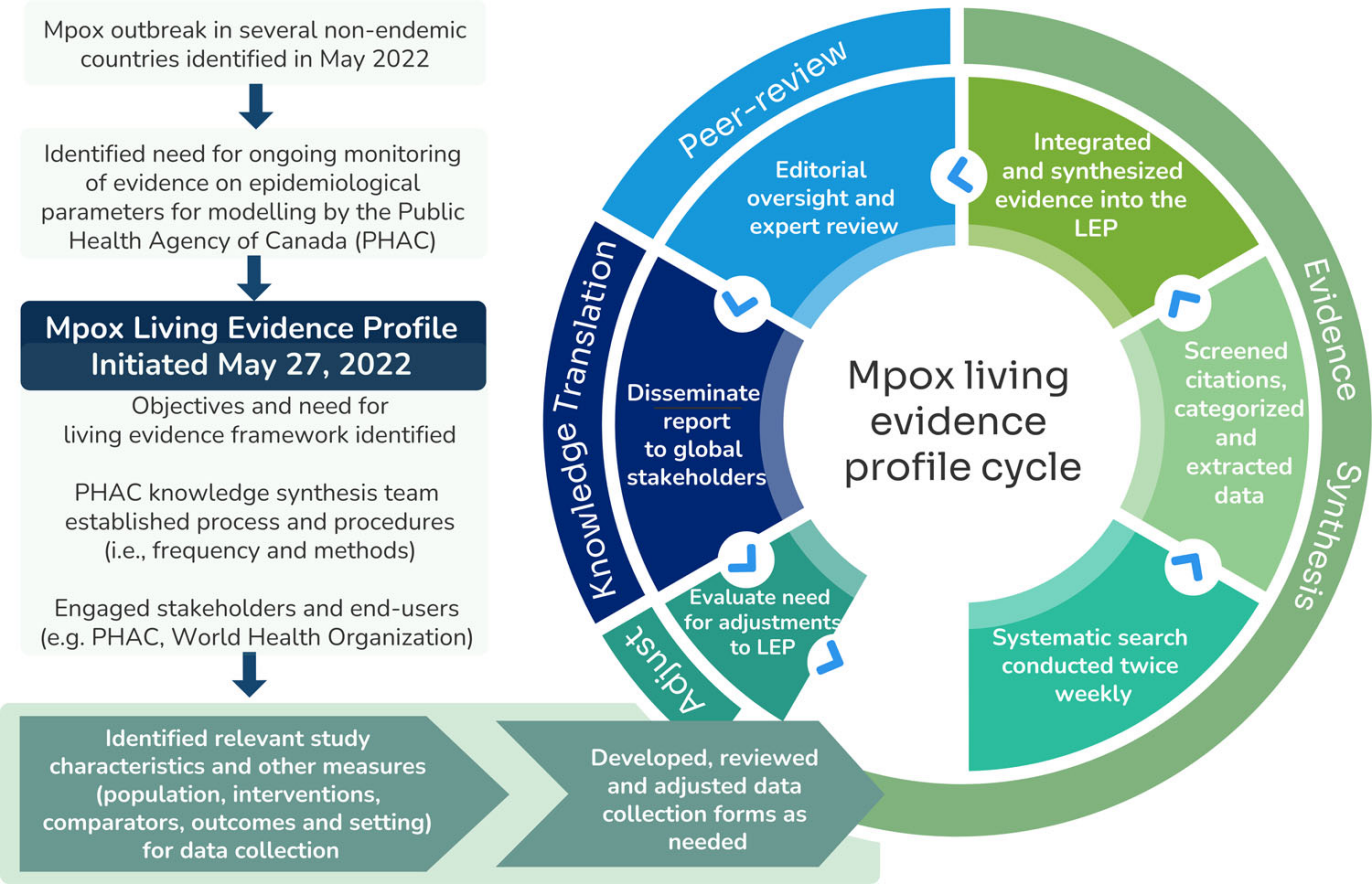
The weekly mpox living evidence profile framework.

There were six main steps within the weekly LEP cycle (Figure 1): twice weekly systematic search of the literature; screening for relevance, categorization, and extraction of data; rapid integration and synthesis of evidence into the LEP; an established editorial process with subject matter experts and accelerated approval process; dissemination of the report to stakeholders; and an evaluation of the need for adjustments (e.g., additional foci and/or frequency) for the next iteration of the LEP. A detailed overview of each step can be found in File S1, a blank framework with instructions and examples (File S2) and a list of foci with definitions (File S3).

### 2.1 Stakeholder engagement

The LEP framework underwent several iterations to incorporate new research on foci not covered in the profile. To support public health decision‐making, stakeholders were consulted on the study characteristics and foci to ensure relevant evidence was optimally organized.

## 3 SEARCH

A comprehensive search was developed by an experienced information specialist and peer‐reviewed by international colleagues (File S1). Pubmed, Scopus, EuropePMC, SSRN, and ArXiv were searched twice weekly from May 31 to December 15, 2022. Grey literature searches of public health government and organization websites were conducted weekly between June 1 to December 15, 2022.

### 3.1 Data management

Citations were collated in an accessible RefWorks (ProQuest) citation database. Distiller SR (Evidence Partners 2022) was used to manage screening and data extraction.

## 4 ELIGIBILITY CRITERIA

Primary research on mpox conducted from April 2022 onward in any language were included in the LEP. Animal models evaluating interventions were included whereas animal models of disease were excluded. Articles not on mpox and non‐primary articles (e.g., reviews and commentaries) were excluded. Historical information from systematic reviews were initially included as a benchmark for the foci of interest but were removed 3 months into the outbreak as there was sufficient new evidence. Case reports with limited utility were removed from the LEP once more robust observational studies accumulated. All citations on mpox were available in the accessible excel dataset that was updated daily with newly extracted data and twice a week with search results.

### 4.1 Study selection and data extraction

To manage the rapid process, title/abstract, full text screening and data extraction were conducted by one reviewer (AB, DA, KP, TC, and LW) and spot checked by a senior reviewer (KP or LW) twice weekly. Disagreements were resolved by consensus or third‐party arbitration.

Extracted study characteristics included: type of report, country of conduct, historical or current outbreak evidence, mpox clade, study design, and population. Evidence was tagged to one or more of 32 pre‐determined foci that aided in grouping together studies by their key outcomes.

### 4.2 Evidence synthesis

New evidence was highlighted in the “What's New” section, organized by foci, and contextualized by providing the study design, country of conduct, sample size, time frame of conduct, and a description of whether the new evidence agreed with what was known from previous LEP versions.

Within the LEP table, the new evidence was integrated with previously synthesized evidence for each foci and highlighted in grey to easily visualize what was added. As evidence accumulated, the narrative synthesis focused on grouping similar studies together into digestible concise summary points, and in some cases this required subcategories to be created.

### 4.3 Certainty of evidence

Formal risk of bias assessments were not conducted due to the volume and rapid nature of the LEP. However, this LEP framework developed a method that required minimal information to convey the overall level of confidence in the evidence based on the contributing study designs, quantity of evidence, and consistency in results for each foci (File S4). Briefly, study designs were used to determine where the evidence was grounded in the hierarchy of evidence[20] and then the volume of literature by study design was considered where five or more decently sized studies measuring the same outcome could be considered to be sufficient evidence to evaluate the consistency in direction and magnitude of the outcome(s) across the studies.

As part of the process, each time a new LEP was compiled, one reviewer assessed the level of confidence for each foci (LW or KP) and a second reviewer verified (LW or KP). Disagreements were discussed and resolved.

### 4.4 Internal‐peer review and dissemination

The LEP underwent editorial internal peer‐review by subject matter experts and a science to policy review before approval by the Agency. Knowledge mobilization included mailing lists that grew through knowledge sharing within the network to reach interested decision‐makers and researchers in health organizations and academia globally (File S1).

## 5 RESULTS

### 5.1 Literature surveillance and management

**Figure 2:**
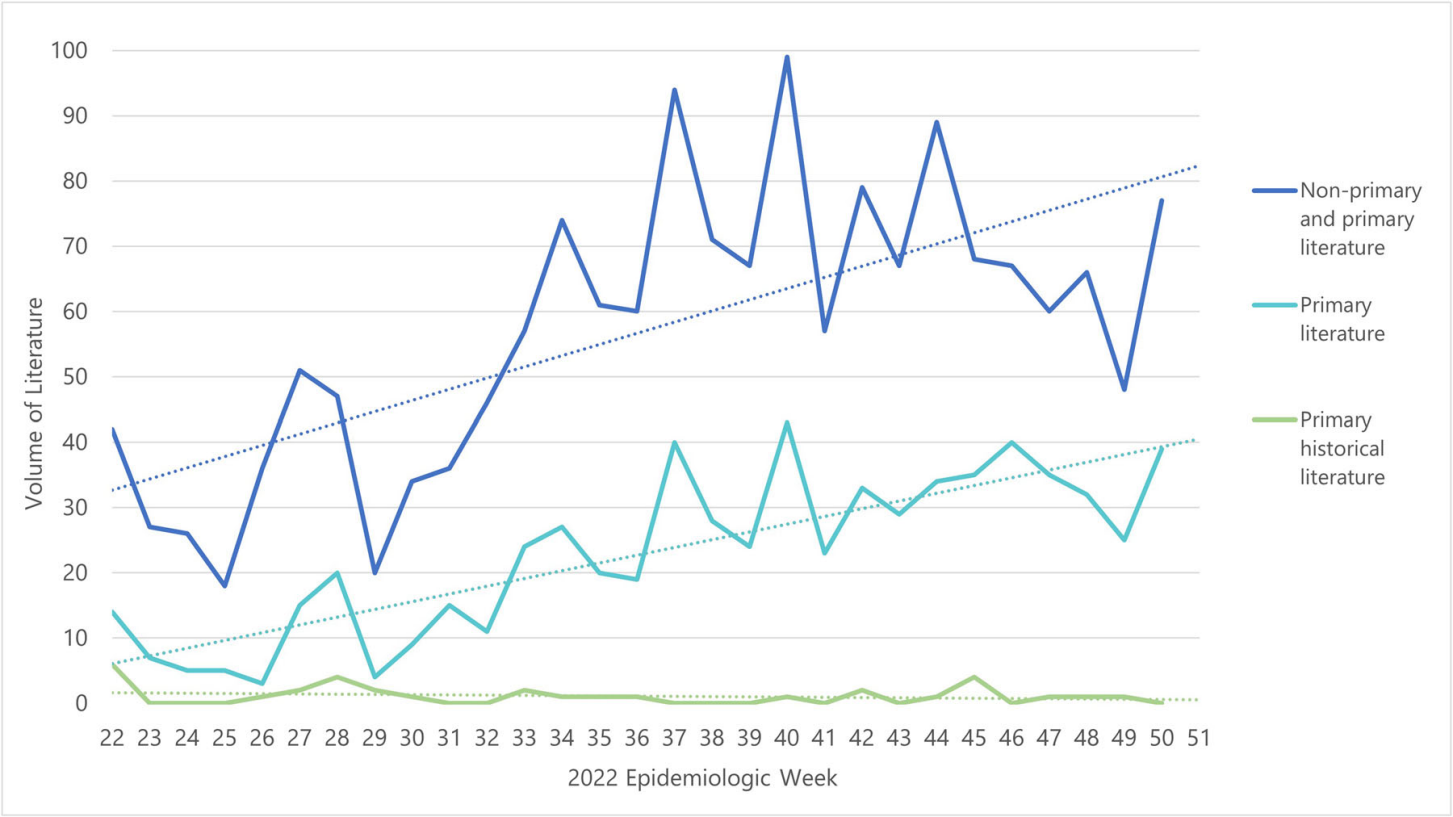
Weekly volume of primary and nonprimary mpox literature from May 31 to December 15, 2023.

The volume of non‐primary and primary literature on mpox increased quickly after the first case in May 2022 (Figure 2). The twice weekly search identified a mean of 57 (range: 18–99) citations, of which 17 (range: 3–43) were classified as relevant mpox primary research. The first search was backdated to April 14, 2022 and retrieved 20 citations, of which two reported evidence from the 2022 outbreak and four reported historical evidence.

**Figure 3:**
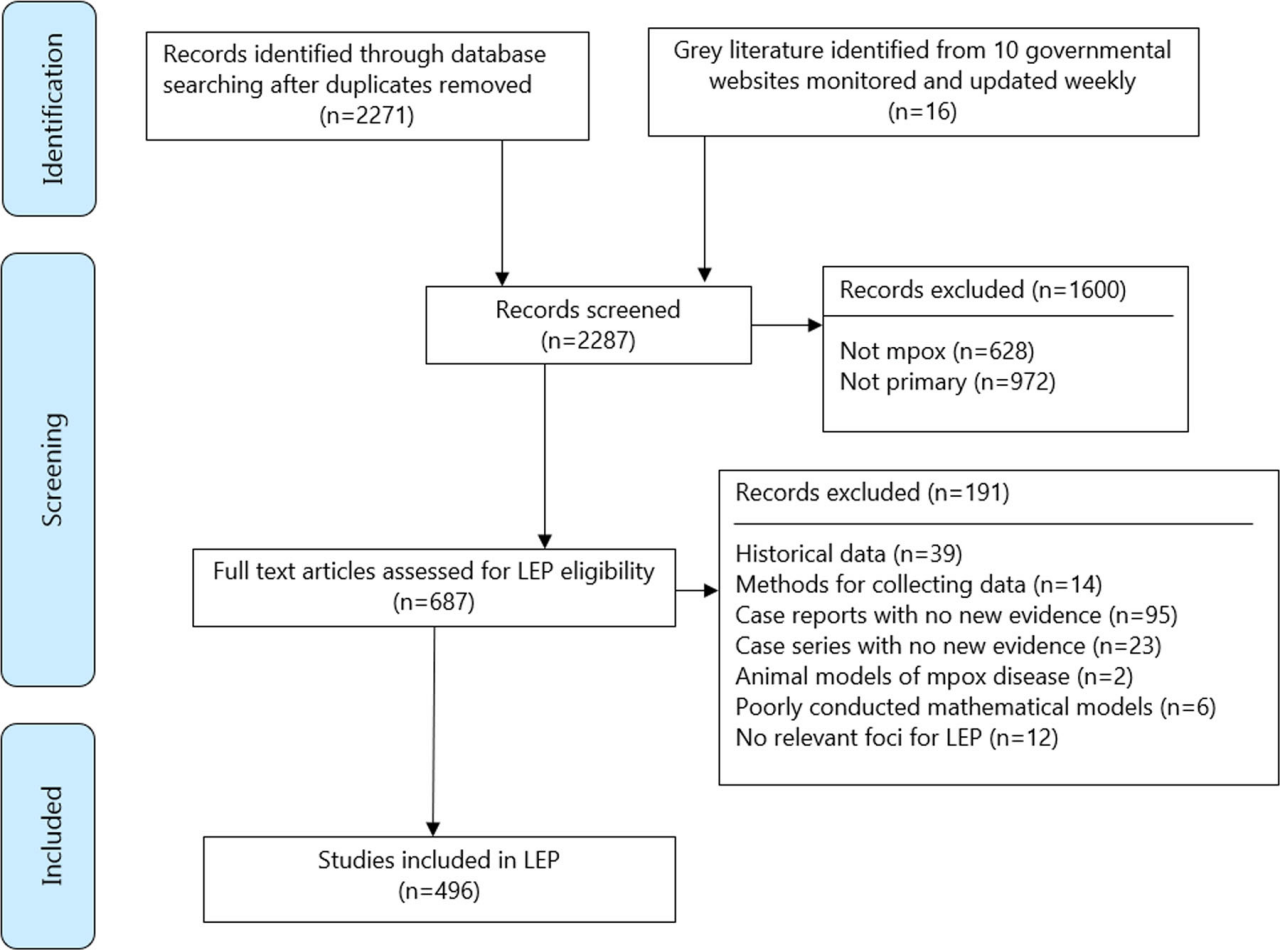
PRISMA flow diagram of the 2287 citations evaluated during the first 6 months of the outbreak.

As of December 15, 2022, 2271 unique citations were captured by the searches and 16 reports, most of which were living reports, from 10 governmental websites (Figure 3). Of these citations, 1600 were excluded from the LEP as they either did not report on mpox or were non‐primary research. A total of 687 primary studies were assessed at full text for eligibility and 496 were included in the final LEP.

The logistics of integrating 3–43 studies into the LEP weekly was challenging despite having a team dedicating the equivalent of one full‐time and three part‐time human hours of work to the project. After the first two months of producing the LEP, there was considerable time dedicated to the on‐going assessment of evidence for each outcome and synthesis to effectively communicate the evidence and its certainty. For example, once there were analytical observational studies (i.e., 2–3 months into the outbreak) for a foci, the integration of case reports and small case series into the LEP were reduced to highlighting them only in the “What's New” section unless they provided novel findings that were not consistent with the synthesis. Strategies, such as using sub‐categories (e.g., by country and by diagnostic test type) within foci helped to improve the utility of the synthesis.

### 5.2 Characteristics of included studies

The characteristics of studies included in the final LEP are reported in Table 1 and are considered to be a good representation of the study designs expected at the beginning of a public health event. Six months into the outbreak, the primary research in the mpox database was comprised of 45% (222/496) peer‐reviewed journal articles, 24% (119/496) preprints that were not published yet, 14% (68/496) letters to the editor, 11% (56/496) short communications, 3% (14/496) commentary/correspondence articles, 3% (16/496) reports, and 0% (1/496) conference papers. Of the peer‐reviewed published studies 15% (73/496) were originally captured as a preprint. Forty‐eight percent (237/496) of studies were conducted in Europe, 26% (1/496) in North America, 18% (88/496) in Asia, 5% (26/496) in the Middle East, 5% (23/496) in South America, and 1% (4/496) each in Australia and Africa. The global distribution of research would vary in other public health events depending on where affected populations are located. Most included studies reported only on the 2022 mpox outbreak (96%; 478/496) while the remaining included 2022 outbreak as well as historical evidence (4%; 18/496). High‐risk populations were described in 14% (67/496) of studies: 16 studies in children, 25 in immunocompromised individuals, 22 in health workers, one in pregnant women, and three in a general high‐risk group category.

**Table 1:** Characteristics included studies at six months from the start of the outbreak (*N* = 496).

Category	Description	Number of studies *N* = 496, *n* (%)
Type of report	Commentary/correspondence	14 (3)
	Conference paper	1 (0)
	Letter to the editor	68 (14)
	Peer‐reviewed journal article	222 (45)
	Preprint	119 (24)
	Report	16 (3)
	Short communications	56 (11)
Region of conduct	Europe	237 (48)
	North America	130 (26)
	Asia	88 (18)
	Middle East	26 (5)
	South America	23 (5)
	Africa	4 (1)
	Australia	4 (1)
	Multiple countries	16 (3)
Timeframe of evidence	2022 outbreak	478 (96)
	2022 outbreak with historical data	18 (4)
Clade	Clade I (Formerly Congo Basin)	0 (0)
	Clade II (Formerly West African)	69 (14)
	Clade I and II	24 (5)
	Not Specified but data was from current outbreak	404 (81)
High risk populations:	Children	16 (3)
	Pregnant women	1 (0)
	Immunocompromised	25 (5)
	Healthcare workers	22 (4)
	High risk group not specified	3 (1)
Study design	Case report	85 (17)
	Case series	56 (11)
	Case‐control	2 (0)
	Cluster investigation	7 (1)
	Cross‐sectional	51 (10)
	Diagnostic accuracy studies	56 (11)
	Ecological study	2 (0)
	Expert elicitation	1 (0)
	In silico study	39 (8)
	In vitro study	25 (5)
	Internet analytics	14 (3)
	Mathematical modeling	26 (5)
	Phylogenetic analysis report	29 (6)
	Predictive model	21 (4)
	Prospective cohort	13 (3)
	Retrospective cohort	31 (6)
	Surveillance data analysis	52 (10)
	Animal model	4 (1)

**Figure 4:**
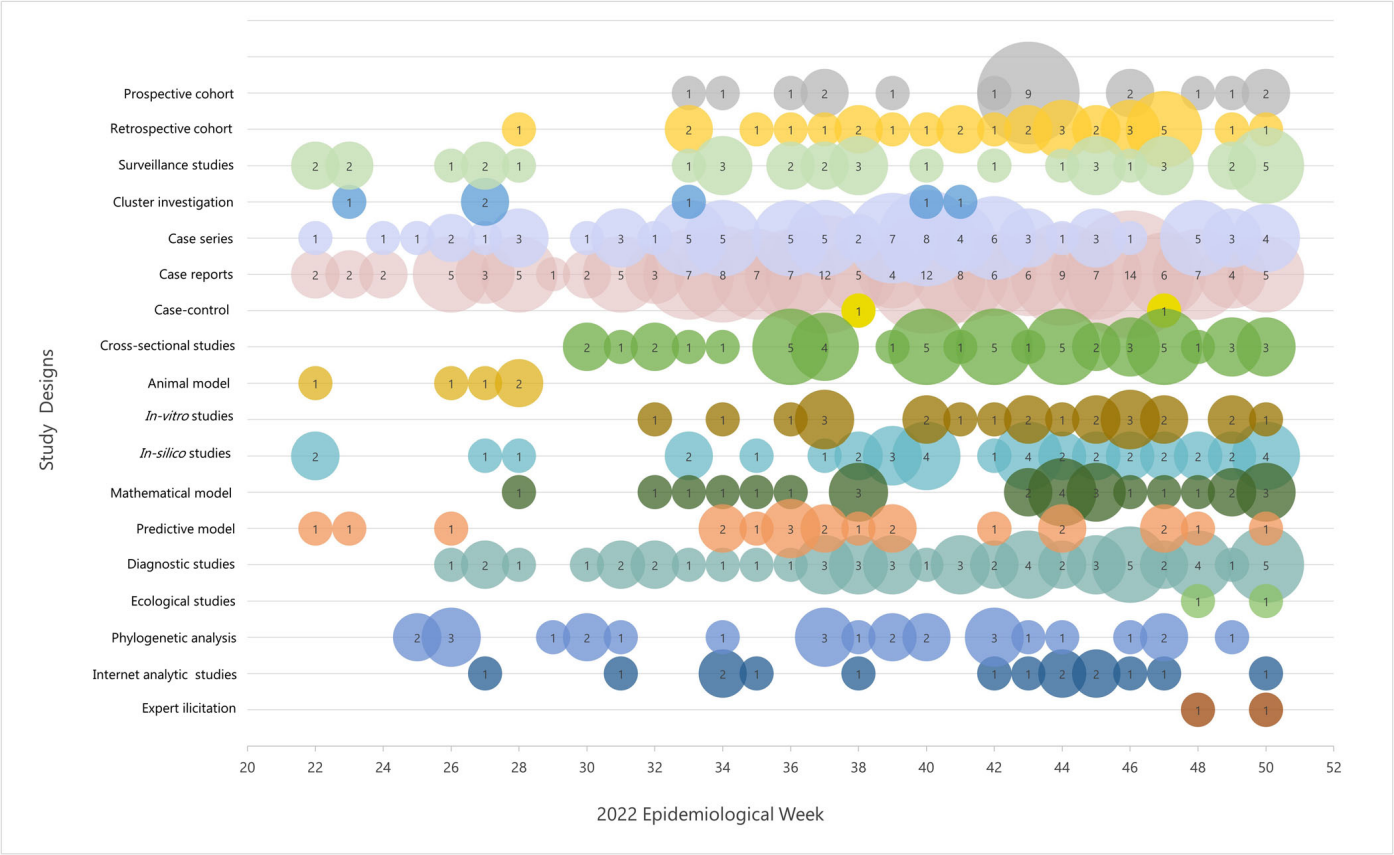
Weekly count of the primary mpox literature by study design from May 31 to December 15, 2023.

The frequency of study designs changed over time with descriptive studies being the most common (28%; 141/496) (Figure 4). In the first six weeks, mainly descriptive studies (e.g., case reports and case series) and surveillance studies were identified. The first retrospective cohort was identified six weeks into the outbreak, but they were not consistently identified until Week 20. At six months, only a few prospective (3%; 13/496) and retrospective cohorts (6%; 31/496) were reported (Table 1). As expected due to the time to design and execute randomized‐controlled trials, none were identified before the final LEP was produced.

### 5.3 Framework for the categorization of LEP foci

**Figure 5:**
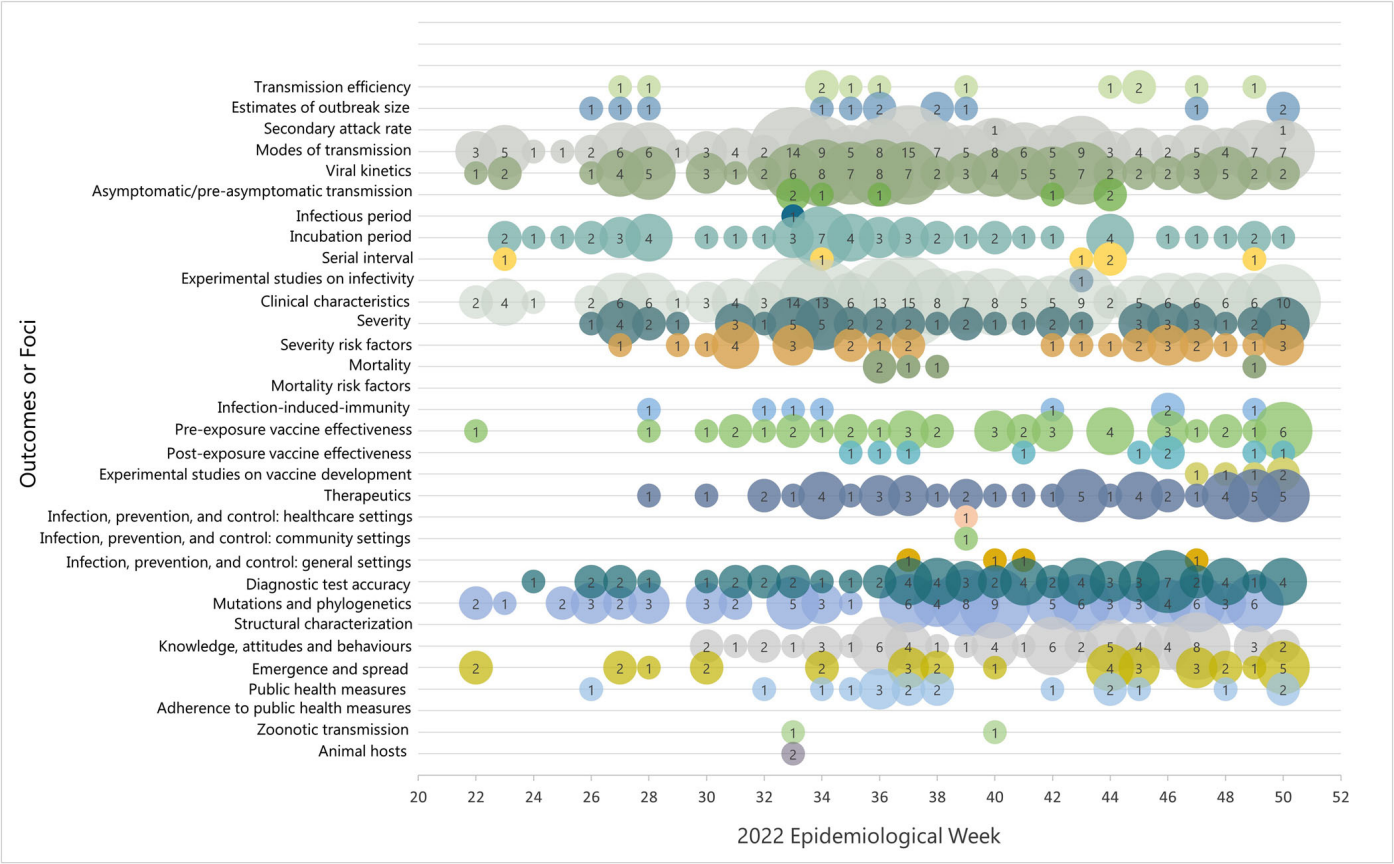
Weekly number primary studies on mpox by the 32 foci reported in the LEP from May 31 to December 15, 2023.

Study outcomes were organized into 32 pre‐determined foci (Figure 5). In the first six weeks of the outbreak the most common foci were modes of transmission, viral kinetics including viral load and viable virus in various samples, incubation period, clinical characteristics, mutations and phylogenetic analyses, and diagnostic test accuracy. By week 18 evidence was reported for 94% (30/32) of the pre‐determined foci. There was no evidence on mortality risk factors and adherence to public health measures. Some foci were rarely reported including infectious period, serial interval, secondary attack rate, mortality and mortality risk factors, infection, prevention and control measures (IPC), postexposure vaccination, and infection‐induced protection.

### 5.4 Certainty of the evidence

In the first six weeks of the outbreak, the evidence informing many foci was sparse and there was low confidence in the results. By week seven, sufficient evidence had accumulated on clinical characteristics from larger analytical epidemiology studies that were consistent with earlier evidence from descriptive studies to upgrade the evidence in the foci to moderate level of confidence. By week 17 the evidence on incubation period, modes of transmission, and severity were also assessed to be sufficient for an upgrade to moderate level of confidence. Serial interval was upgraded at week 29 when the final LEP was produced. Six months into the outbreak, 88% (28/32) of foci were classified as low level of confidence, 13% (4/32) as moderate and none were rated as high due to lack of strong study designs (e.g., controlled trials).

## 6 FREQUENCY OF LEP PRODUCTION

During the mpox outbreak there was a need for weekly updates for the first 12 weeks as the evidence was changing rapidly. After this time the cases were declining, the evolution of the evidence slowed down and the Agency assessed the risk to be low for the general population and well controlled within the high‐risk populations. In consultation with stakeholders, the LEP production was reduced to every other week at week 25 of the outbreak and then was decommissioned on week 29 as the new literature was not changing our overall knowledge, there were few new cases and most response activities were being scaled back. However, in support of several end‐users with on‐going response work, the literature search and excel dataset continued to be updated as of December 2023 to keep apprised of emergent literature. There were 23 LEPs produced between 31 May and 15 December 2022 that spanned the period of the Agency's main outbreak response.

### 6.1 Collaboration and knowledge mobilization

The mpox LEP and dataset were used extensively by outbreak response teams in the Agency involved in modeling, public health measure guidance, rapid risk assessments, and by teams responding to urgent requests for evidence. The outbreak response team at the WHO utilized the LEP reports and excel dataset to support their public health response activities by providing including the addition of an epidemiological parameters (e.g., incubation period and serial interval) summary in the mpox Global Trends report [2]. There were over 60 recipients subscribed to the mpox LEP mailing list who were part of Canadian public health, international organizations, and academia including knowledge synthesis experts, epidemiologist, public health practitioners, and mathematical modeling teams located in Canada, the United Kingdom, the United States, and Australia that utilized the LEP for their response efforts.

## 7 DISCUSSION

This paper describes the development and maintenance of a LEP to inform evidence‐based public health decision making during the first six months of the 2022 mpox outbreak. The LEP framework was inspired by concepts of living systematic and rapid review attributes of continual literature retrieval and rapid synthesis which supported decision making during the COVID‐19 pandemic [15, 21, 22]. The framework was designed to be applied to the unique situation of the first few months of a priority public health event or outbreak where there is the rapid accrual of evidence and an urgent need for timely evidence synthesis that is at most a few days out of date [15]. This dynamic framework allows for small adjustments to the LEP methods at each iteration when new terminology needs to be integrated into the search strategy or for the addition of new foci or reorganization a foci's sub‐categories as evidence accumulates.

The utility and confidence in the evidence evolves rapidly during an outbreak. The early evidence was mostly descriptive and provided details about modes of transmission, clinical characteristics, and incubation period. Other key epidemiological parameters (e.g., infectious period, secondary attack rate, and serial interval) needed for public health response activities and modeling were rarely reported or only sporadically described for cases, but not estimated with measures of variability. By design, the LEP easily conveyed the knowledge gaps and limitations of existing evidence in addition to the available evidence which was also important information for public health response.

There are several factors that influence timing and quality of evidence produced during an outbreak. These include the nature of the outbreak, the ability of public health to track cases (both capacity and influence by the disease), whether there are surveillance/reporting systems available to capture data, the geographic region of the outbreak, and the urgency of the response. Considering normal research timelines mpox research was rapidly produced, but the outbreak had already peaked when analytical epidemiology studies with better estimates and measures of variability became available. Thus, initial response efforts relied on the small body of historical evidence and early descriptive studies. Strategies to produce analytical studies (observational or trials) faster would have a large impact on response activities. There was a dearth of data for some mpox epidemiological parameters and not others which may stem from the challenges of obtaining accurate contact data from mostly intimate contacts during the contact‐tracing investigations [23] and clinical trials on the course of infection can not be set up quickly or are impossible to conduct during an outbreak [24].

Important and on‐going considerations of conducting any living evidence synthesis is the frequency and timing of updates (e.g., how quickly the topic is evolving and the needs of the end‐users), how to communicate changes in the methodology between each iteration, how to communicate updates at a frequency not conducive to the peer‐review process, how to communicate whether results and conclusions have changed, the logistics of executing and maintaining a living evidence synthesis, and when to decommission [15, 21, 25, 26, 27]. The LEP was developed to be utilized during the early phase of an outbreak (e.g., the first 3–6 months) as it addresses some of the challenges related to conducting other evidence syntheses on a rapidly changing topic. The weekly LEP cycle was at the human resource capacity of the team and resources allocated for peer‐review and approvals by the Agency during the outbreak. Human resources to produce and disseminate an LEP is a challenge as scaling up may not be feasible. Decommissioning required consultation with stakeholders, we suggest initiating the dialog when the utility of the LEP starts to decline because new evidence does not change our knowledge, the outbreak is coming under control, and/or response activities are being scaled back.

Based on feedback from stakeholders, the mpox LEP played a critical role in the Agency and global public health response activities (e.g., mpox rapid risk assessment and modeling activities), decision‐making, and the WHO mpox global trends report [2]. There were several key reasons the mpox LEP had far‐reaching use during the public health response; including the weekly cycle of production, collection of foci that are of interest to a diverse range of decision‐makers, and the strategy of highlighting new evidence in the context of what is already known. The LEP was founded on principles of Open Science to ensure that the synthesized information, dataset and sources were easily accessible, updated in a timely manner, reliable, and trustworthy for decision‐makers [28]. The early awareness and dissemination of the LEP and excel dataset across established networks of end‐users developed during the COVID‐19 and mpox outbreaks were key to its' broad use. The initial networks that were invited to the LEP mailing list were groups that had previously exchanged information with the Agency during the COVID‐19 response. The predictable cycle of the LEP and the consistency of the product provided timely, usable evidence that could be integrated into secondary workflows to maximize efficiency and minimize duplication of efforts globally. The application of the LEP framework to the mpox outbreak is an excellent example of coordinated efforts globally to reduce duplicative knowledge synthesis efforts [21] and mitigate research waste [29].

### 7.1 Strengths, limitations, and future improvements

A comprehensive search strategy was implemented; however, it is possible some relevant papers were omitted. The team included individuals with epidemiological, infectious disease, and knowledge synthesis methodological expertise as well as recent experience conducting daily literature searches, categorization of evidence, and rapid evidence briefs during the COVID‐19 pandemic which contributed to the timely conduct and quality of the LEP [17]. Furthermore, we utilized the infrastructure that was in place for editorial oversight and dissemination of evidence syntheses established during the COVID‐19 pandemic.

This framework faces several limitations which are unique challenges associated with rapidly evolving evidence and the process established for the LEP. A single reviewer screened and extracted data while a second experienced reviewer spot‐checked. We suspect the bias to be minimal as the LEP underwent rigorous peer‐review and experienced reviewers have been shown to accurately screen articles with negligible impact on the overall findings of a rapid review [30].

The method to assess overall level of confidence for each foci is limited, guideline thresholds for upgrading the certainty of evidence had an element of subjectivity particularly for how much variability was tolerable for results to be considered consistent. However, the use of established risk of bias assessment tools was precluded as there was insufficient time to formally assess articles. Further research is needed to investigate or develop metrics to evaluate confidence in the evidence within the LEP cycle.

Preprints were included due to the urgent need for timely information. They have been shown to potentially have errors in the dataset that may get resolved before publication, may be less complete and/or have changes to the spin in the conclusions [31] Most preprint to print changes have also not resulted in meaningful changes to the conclusions of the study [32].

The dissemination of the LEP was also limited to the Agency's networks given that the Agency currently does not have a mechanism to rapidly publish accessible documents online. Improving the discoverability is a priority for future outbreaks.

Improvements in efficiency may be realized as artificial intelligence tools evolve given several LEP steps including the search, screening, and data extraction could become more automated which would alleviate some of the human resources required to maintain a LEP [33]. Use of interactive living dashboards where evidence could be automatically updated would allow the curated evidence and synthesis to be disseminated in real‐time and discovered by additional end‐users that were not connected to our network.

## 8 CONCLUSIONS

This novel LEP employed during the 2022 mpox outbreak demonstrates the utility of systematically identifying and organizing rapidly emerging evidence in an on‐going cycle during the beginning of a public health emergency for a wide range of end‐users. A key attribute of the LEP was that evidence was organized in a way that decision‐makers could quickly identify new information in the context of what was already known and where knowledge gaps continued to exist. The impact of the LEP on response efforts is largely attributable to early awareness and sharing of results across established networks, which was key to engaging stakeholders, reducing duplicative efforts, and mitigating research waste. This framework can be tailored and implemented to other public health emergencies to support response activities.

## AUTHOR CONTRIBUTIONS

**Kusala Pussegoda**: Conceptualization; data curation; formal analysis; investigation; methodology; project administration; validation; visualization; writing—original draft; writing—review and editing. **Tricia Corrin**: Data curation; writing—review and editing. **Austyn Baumeister**: Data curation; writing—review and editing. **Dima Ayache**: Data curation; writing—review and editing. **Lisa Waddell**: Conceptualization; data curation; methodology; project administration; supervision; validation; writing—original draft; writing—review and editing.

## CONFLICT OF INTEREST STATEMENT

The authors declare no conflict of interest.

## PEER REVIEW

The peer review history for this article is available at https://www.webofscience.com/api/gateway/wos/peer-review/10.1002/cesm.12044.

## Supporting information

Supplementary information.

Supplementary information.

Supplementary information.

Supplementary information.

## Data Availability

This article has earned an Open Data badge for making publicly available the digitally shareable data necessary to reproduce the reported results. The data that support the findings of this study are openly available in The Open Science Framework at https://osf.io/yu3td/files/osfstorage/65b1705aebe5e606c3f5f16b.
