# Supplementary material for: Methods for conducting a living evidence profile on mpox: An evidence map of the literature
Source: Cochrane Evid Synth Methods. 2024 Feb 22;2(2):e12044. doi: 10.1002/cesm.12044 (PMC11795934; doi:10.1002/cesm.12044)
Supplement: Supplementary file 1 — Supplementary information. [file CESM-2-e12044-s002.docx]

Supplementary Material 1

## A LEP was initiated on May 27, 2022 after evaluating the need for up-to-date evidence by stakeholders within the Public Health Agency of Canada, herein referred to as Agency. The LEP framework was developed by the Agency and utilized structured and reproducible knowledge synthesis methodologies (1, 2). The content and organization of the evidence in the LEP was informed by hazard profiles from food safety risk assessment, epidemiological parameters needed for infectious disease modelling, consultation with public health experts and knowledge of the important types and categories of studies that were likely to be published given the team’s experience with infectious diseases and emerging evidence during the COVID-19 pandemic (3-5). During the COVID-19 pandemic, the knowledge synthesis team at the Agency developed the COVID-19 literature surveillance framework where the team developed expertise in searching and disseminating literature daily to over 200 recipients and organizations(3). An email was sent daily with a Microsoft Word template line list of each new article, foci(s) covered and summary. The LEP utilized this continuous framework for literature searching and dissemination and applied it the mpox outbreak. For the mpox LEP, we refined foci/topic areas to those relevant for mpox and subsequently categorized and synthesized evidence under each foci. Evidence in each foci was assessed for certainty, new studies were highlighted and the LEP underwent an internal peer-review. An overview of each step in the LEP framework is described below.Stakeholder engagement

The novel LEP framework was developed by the knowledge synthesis team at the Agency and underwent several iterations throughout its development and initial production. Study characteristics and foci were selected and adjusted in consultation with methodological, clinical and infectious disease modelling experts as well as collaborators from the WHO to ensure relevant evidence was organised to support national and global public health decision-making. As this was a dynamic synthesis framework, additions to the data collection form were needed over time to incorporate new research on foci not yet covered in the profile.

# **Search**

A comprehensive search was developed by an experienced information specialist in consultation with the review team and peer-reviewed by international colleagues. Pubmed, Scopus, Europe PMC, SSRN and ArXiv were searched twice weekly from 31 May to 15 December 2022 to identify new preprint and published literature on mpox. Search terms used included: Monkeypox*, Monkey pox, Monkey orthopox*, mpox, Simianpox*, Simian pox*, Simian orthopox, MPXV, Monkeypox virus, Variole du singe, orthopoxvirose simienne, Variole simienne. Studies between 14 April 2022 and 15 December 2022 were included.

Grey literature searches were conducted weekly between 1 June to 15 December 2022 to monitor reports by WHO, European Centre for Disease Prevention and Control, Public Health Agency of Canada, United States Centre for Disease Control and Prevention, United Kingdom Health Security Agency, other government public health websites, and official reports on mpox cited in the media.

The mpox search algorithm is adapted to each database:

Pubmed

*Monkeypox[All Fields] OR Monkeypox*[All Fields] OR "Monkey pox*"[All Fields] OR "Monkey orthopox*"[All Fields] OR Simianpox*[All Fields] OR "Simian pox*"[All Fields] OR "Simian orthopox*"[All Fields] OR MPXV[All Fields] OR Monkeypox[MeSH Terms] OR Monkeypox virus[MeSH Terms] OR "Variole du singe"[All Fields] OR "orthopoxvirose simienne"[All Fields] OR “Variole simienne”[All Fields] OR “mpox” [All Fields]*

Scopus

*TITLE-ABS-KEY (Monkeypox OR “Monkey pox” OR “Monkey orthopox” OR Simianpox OR “Simian pox” OR “Simian orthopox” OR MPXV OR “Monkeypox virus” OR “Variole du singe” OR “mpox” OR “orthopoxvirose simienne” OR “Variole simienne”)*

EuropePMC

(Title:(Monkeypox) OR Title:("Monkey pox") OR Title:("Monkey orthopox") OR Title:(Simianpox) OR Title:("Simian pox") OR Title:("Simian orthopox") OR Title:(MPXV) OR Title:("Variole du singe") OR Title:("orthopoxvirose simienne") OR Title:("Variole simienne") OR Title:(“mpox”) OR Abstract:(Monkeypox) OR Abstract:("Monkey pox") OR Abstract:("Monkey orthopox") OR Abstract:(Simianpox) OR Abstract:("Simian pox") OR Abstract:("Simian orthopox") OR Abstract:(MPXV) OR Abstract:("Variole du singe") OR Abstract:("orthopoxvirose simienne") OR Abstract:("Variole simienne") OR Abstract:(“mpox”)) AND (SRC:PPR)

SSRN and arXiv

Searched using the following key words: Monkeypox, Simianpox, Variole du singe and Variole simienne, and mpox

## Data management

Citations from the bibliographic databases and grey literature were collated in Endnote (Clarivate, Philadelphia, Pennsylvania, USA). De-duplication of articles was first conducted in Endnote and then transferred to RefWorks (ProQuest) as an accessible reference management platform for end-users. A final check for duplicates was also conducted when search results were uploaded to the online Systematic Review management software program, Distiller SR (Evidence Partners 2022) where the screening and data extraction steps of the LEP process were managed.

# **Eligibility criteria**

For inclusion in the LEP, the citation had to be primary research on mpox conducted from April 2022 onward in any language. Animal models evaluating interventions~~vaccine candidates~~ were ~~also~~ included whereas animal models of disease were excluded. Articles not on mpox and non-primary articles (e.g., reviews and commentaries) were excluded.. Initially, historical information from systematic reviews were included as a benchmark for the foci of interest, but three months into the outbreak (September 7, 2022) there was sufficient evidence from the 2022 outbreak so this data was removed. Similarly, on September 7, 2022 descriptive studies (e.g., case reports) with few observations were also removed from the narrative synthesis and only highlighted each week in the “What’s New” section of the LEP as they had limited utility once more robust observational studies started to accumulate. Exceptions were made for unique or contradictory evidence. All citations on mpox including those included in the LEP and those excluded based on the eligibility criteria were made available in a accessible excel dataset to end-users that was updated daily with newly extracted data and twice a week with search results.

**Study selection**

To manage the rapid process efficiently, title/abstract and full text screening were conducted by one reviewer (AB, DA, KP, TC, LW) and spot checked by a senior reviewer (KP or LW) twice weekly. The screening form was pilot-tested and reviewed with the review team to ensure consistency. Disagreements were resolved by consensus or third-party arbitration. Citation screening was conducted to identify citations on mpox and classify the literature as primary or non-primary research.

## Data extraction

A pilot test of the data extraction form was pre-tested with the review team on 50 papers and adjustments were made to improve the clarity of the form. Data extraction was performed by one reviewer (AB, DA, KP, TC, LW) and spot-checked by a senior reviewer (KP or LW). Disagreements were resolved by consensus. Extracted study characteristics included: type of report (e.g., peer-reviewed article, preprint, letter to the editor), country of conduct, historical or current outbreak evidence, mpox clade, study design and population. Evidence from each study was categorized and tagged to one or more of 32 predetermined foci that aided in grouping together studies by their key outcomes and relevant data was extracted. Results from studies were incorporated in the LEP. Data extracted from preprints were updated once the peer-reviewed publication became available. The excel dataset was updated daily with newly extracted data and twice a week with search results so end-users had real-time access to emerging evidence.

## Evidence synthesis

Based on the anticipated volume of mpox studies and the weekly cycle of the LEP, evidence was narratively synthesized within each foci in a table within the LEP. As new evidence emerged, it was organized by foci and highlighted in the “What’s New” section of the LEP. Evidence was contextualized by providing the study design, country of conduct, sample size, time frame of conduct and a description of whether new evidence agrees with what was known from previous LEP versions.

Within the LEP table the new evidence was integrated with previously synthesized evidence for each foci and highlighted in grey to easily visualize the new evidence that was added. As evidence accumulated, the narrative synthesis focused on grouping similar studies together into digestible concise summary points, and in some cases this required subcategories to be created.

## Certainty of evidence

Formal risk of bias assessments were not conducted due to the volume and rapid nature of the LEP. However, this LEP framework developed a method that required minimal information to convey the overall level of confidence in the evidence based on the contributing study designs, quantity of evidence and consistency in results for each foci(see supplementary material s4). Briefly, study designs were used to determine where the evidence was grounded in the hierarchy of evidence the evidence (6) and then the volume of literature by study design was considered where five or more decently sized studies measuring the same outcome could be considered to be sufficient evidence to evaluate the consistency in direction and magnitude across the studies.

Descriptive studies such as case reports and case series provide information that can be used to generate hypotheses for further study but are considered very low confidence evidence. Many analytical observational studies (case control, cross-sectional or retrospective cohorts) were considered low confidence. Predictive models similarly are considered very low confidence evidence because the generalizability of each model can be context specific and sensitive to the data used to parameterize the model. However, using the models to compare scenarios of varying assumptions and interventions is extremely valuable for decision-making and thus this information was included in the LEP with a caution statement to remind readers that models are primarily meant to explore different possible scenarios. The *in-silico* studies are used to generate hypotheses and the results of these studies would need to be tested with *in vitro* and *in vivo* study designs, thus are considered very low confidence evidence.

For most outcomes evidence accumulated from descriptive studies to analytical epidemiology studies that were mainly retrospective and prospective cohorts. Studies were upgraded over time to moderate certainty when foci in the LEP had prospective cohorts contributing evidence and there was consistent findings across studies. This was expected considering the LEP framework was applied to an emergent situation and would likely hold true across other public health events.

As part of the process, each time a new LEP was compiled, one reviewer assessed the level of confidence for each foci (LW or KP) and a second reviewer verified (LW or KP). Disagreements were discussed and resolved.

## Internal-peer review and dissemination

Editorial internal peer-review by subject matter experts and a science to policy review prior to obtaining approval by the Agency. Knowledge mobilization of the LEP was coordinated by the Office of the Chief Science Offer of the Agency. A mailing list was created and users/organizations signed-up to receive the LEP and excel dataset each week through email. The email included a template describing what the LEP is and included the PDF of the LEP for that week as well as the links to an open access excel dataset accessible through Google Drive and RefWorks database containing all the primary and non-primary articles. The mailing lists grew through knowledge sharing within the networks that our Agency established during the COVID-19 pandemic as well as during the mpox outbreak. The collaboration with the WHO was a key component for the knowledge and utilization of the LEP with their global connections. Each iteration of the LEP was disseminated to over 60 direct recipients and organizations including provincial, national and international health organizations and academic institutions.. The Agency also disseminated the profile weekly through networks developed by the Office of the Chief Science Office. Some examples of institutions that had subscribed to the mailing list include the Public Heath Agency of Canada, the British Columbia Centre for Disease Control, Institut national de santé publique du Québec, the WHO, Ministry of Health and Social Services, Liverpool School of Tropical Medicine, Centre for Disease Control, Monash University, London School of Hygiene and Tropical Medicine,

References:

1. Cochrane Colloboration. Guidance for the production and publication of Cochrane living systematic reviews: Cochrane Reviews in living mode. 2019.

2. Tricco AC, Lillie E, Zarin W, O'Brien K, Colquhoun H, Kastner M, et al. A scoping review on the conduct and reporting of scoping reviews. BMC Med Res Methodol. 2016;16:15.

3. Corrin T, Ayache D, Baumeister A, Young K, Pussegoda K, Ahmad R, et al. COVID-19 literature surveillance-A framework to manage the literature and support evidence-based decision-making on a rapidly evolving public health topic. Can Commun Dis Rep. 2023;49(1):5-9.

4. Food and Agriculture Organization of the United Nations. Food Safety Risk Management 2017 [Available from: <https://www.fao.org/documents/card/en/c/I8240EN/>.

5. Ogden NH, Fazil A, Arino J, Berthiaume P, Fisman DN, Greer AL, et al. Modelling scenarios of the epidemic of COVID-19 in Canada. Can Commun Dis Rep. 2020;46(8):198-204.

6. PurdueGlobal. Evidenced Based Practice Pyramid of Resources 2023 [Available from: <https://library.purdueglobal.edu/ebn/ebpyramid>.
