# Supplementary material for: Methods for conducting a living evidence profile on mpox: An evidence map of the literature
Source: Cochrane Evid Synth Methods. 2024 Feb 22;2(2):e12044. doi: 10.1002/cesm.12044 (PMC11795934; doi:10.1002/cesm.12044)
Supplement: Supplementary file 2 — Supplementary information. [file CESM-2-e12044-s003.docx]

Living evidence profile on the [Insert outbreak/event name]

Highlights up to [INSERT MONTH, DAY, YEAR]

**Table of contents**

[Background 1](#_Toc1183095263)

[What’s new [INSERT MONTH DAY-MONTH DAY, YEAR] 1](#_Toc2108223642)

[Overview of the evidence 2](#_Toc1406415857)

[Evidence table 4](#_Toc1806986480)

[Table 1: Evidence on the [INSERT OUTBREAK/EVENT NAME] (n=) 4](#_Toc879232918)

[Methods 7](#_Toc896570397)

[Acknowledgements 7](#_Toc1291233924)

[References 7](#_Toc1440846755)

# Background

**As of [Month Day, Year], what is known about the outbreak globally in terms of transmissibility and spread, clinical severity, protective immunity, treatment effectiveness and the impacts of public health measures****?**

[INSERT BACKGROUND INFORMATION ON OUTBREAK/EVENT]

This living evidence profile on [INSERT OUTBREAK OR EVENT] is designed to capture information on the epidemiological parameters including transmission rates, clinical outcomes of severity and mortality, affected age groups, protective immunity, therapeutics, diagnostic/detection tests, genomics, impacts of public health measures and knowledge and attitudes towards [INSERT DISEASE NAME]. The literature list of [INSERT OUTBREAK EVENT] evidence published since [ENTER DATE] is available in the [INSERT LINK TO DATASET]. Citations may be accessed in the citation management software RefWorks for the primary research used in this profile [INSERT LINK TO REFWORKS OR OTHER ACCESSIBLE CITATION DATABASE].

# What’s new [INSERT MONTH DAY-MONTH DAY, YEAR]

There were [INSERT NUMBER OF STUDIES] studies published on the [INSERT OUTBREAK/EVENT NAME] between [INSERT MONTH DAY-MONTH DAY, YEAR], including [INSERT TYPE OF STUDY DESIGN AND NUMBER OF EACH DESIGN IDENTIFIED IN CURRENT LEP CYCLE]. Note: case reports that do not offer notable findings will be highlighted in the “what’s new” section but will not be included in the evidence table below. The evidence from these studies can be found in the accessible citation database [OPTIONAL, ADJUSTED AS REQUIRED].

**[INSERT OUTCOMES AND SYNTHESIS OF RESULTS (see examples indicated in dark blue below)]**

**Transmissibility**

Serial interval and reproduction numbers

- A study from the Netherlands up to September 12, 2022 analyzed data on infector-infectee pairs (109 total pairs, 34 pairs with reliable data).^1^ The best estimate of serial interval from the reliable paired data was mean 10.1 days (95%CrI 6.6-14.7 and SD 6.1 days (95%CrI 4.6-8.0)). This was longer than the crude data from all 109 pairs of 6.3 days (SD 6.1). From the best estimate of serial interval, the reproduction number was estimated to be 1.3-1.6 in June 2022.^1^

Incubation period

Nine new studies agree with previous studies that estimated the mean incubation period was 7-9.6 days with a range of 2-21 days ([Table 1](#_Table_1:_Evidence)).

- A study of data from the Netherlands up to September 12, 2022 identified 18 pairs with in-depth epidemiological investigations and estimated the incubation period was a mean 8.1 days (SD 4.4).^1^
- Using data from a previously published prospective cohort study in Spain (May 11-June 30, 2022)^2^ an analysis of gbMSM with receptive anal contact vs gbMSM without receptive anal contact vs. non-gbMSM contact estimated crude incubation periods of 8.0, 7.0 and 6.0 days, respectively.^3^ Caution interpreting the differences in incubation periods across the sub-groups as the study did not report the measure of variability for these estimates.
- The incubation period reported in six case reports and a retrospective cohort was 2-14 days across studies.^4,5,6,7,8,9,10^

**Clinical data**

Severity

The proportion of cases hospitalized range across studies from 1-14%, and main reasons for hospitalization include isolation and treatment for pain or complications, including bacterial superinfection ([Table 1](#_Table_1:_Evidence)).

- Five new case series ^11,12,13,14,15^, four case reports ^16,17,18,19^ and a case-control study ^20^ describe cases hospitalized for clinical management ^11,12,13,16^, worsening of lesions ^14,15,18^, isolation precautions ^13,19^ severe proctitis ^15,17,20^ and severe disease^11^.

Notable studies reporting severity:

- In a retrospective cohort of cis gender and transgender women and non-binary individuals conducted across 15 countries between May – October 2022, 17 of 136 (13%) women (15 cis gender women and non-binary individuals and two trans women) were hospitalized and the risk of hospitalization was the same regardless of HIV status ^21^.

# Overview of the evidence

From [INSERT Month Day, Year to Month Day, Year], there were [ENTER NUMBER OF PRIMARY STUDIES] primary studies published on the [INSERT DISEASE] outbreak and [ENTER NUMBER OF STUDIES] studies included in the evidence profile. The studies include [INSERT NUMBER AND TYPE OF STUDY DESIGNS].

The evidence from the observational study designs is frequently at high risk of bias due to missing information, selection bias and confounding factors. Descriptive studies such as case reports and series provide information that can be used to generate hypotheses for further study, but frequently lack the control group needed for analytics. Predictive models should be used with caution as the models are based on scenarios given a certain context and are parameterized using available observational data at the time of model construction. The applicability of a model's findings will depend on the suitability these factors and should be used with caution. The *in-silico* studies are used to generate hypotheses that are then tested with more rigorous study designs; thus, these results are considered preliminary. For this profile, no formal risk of bias assessment was conducted for each study. A summary indication of the level of confidence of the evidence was given for each category in the LEP based on the study designs, number of studies and agreement across studies in the outcome direction and magnitude [SEE SUPPLEMENTARY S3].

Overall, the current confidence in the evidence on [OUTBREAK] is [INSERT LEVEL OF CONFIDENCE]. Additional studies, analyses and reporting of real-world evidence from the current outbreak are needed to provide data on [INSERT KEY KNOWLEDGE GAPS CREATING UNCERTAINTY IMPORTANT FOR DECISION MAKING ON THE OUTBREAK E.G., *transmission efficiency, secondary attack rates, infectious period, viral load, risk factors for case severity and effectiveness of public health measures, as well as the nature and extent of protective immunity to increase our knowledge and confidence in the summary results for all outcomes*.]

Categories of evidence in the table below include: [NOTE, THE MAIN CATEGORIES BELOW CAN BE EXPANDED TO INCLUDE SUB-CATEGORIES FOR FOCI WITH A LOT OF EVIDENCE AND SUB-CATEGORIES CAN BE REMOVED IF THEY ARE NOT APPLICABLE TO THE OUTBREAK- SEE SUPPLEMENTARY S2 FOR DEFINITIONS OF EACH FOCI].

**Transmissibility** includes changes in transmission efficiency, serial interval (time interval between the onset of symptoms in the primary and secondary case), secondary attack rates, viral load (PCR Ct value is often used as a proxy for viral load with lower Ct value indicative of higher viral load), incubation period, infectious period, estimates of selective advantage and *in silico* studies on cell infectivity.

**Clinical severity** includes proportion of infections that are symptomatic, proportion of infections that develop severe disease e.g., hospitalizations, intensive care unit (ICU), mechanical ventilation and mortality, as well as risk factors for severe disease or mortality. Note, for severe disease or mortality it is important to highlight data on vulnerable populations when reported e.g., people who are pregnant or immunocompromised.

**Protective immunity** includes changes to protection from prior infection, vaccine efficacy/effectiveness in humans, and animal models, *in vitro* or *in silico* experiments on protective immunity. The inclusion of the latter research will depend on the state of the evidence and can be adjusted as evidence evolves.

**Therapeutics** studies on treatments for mpox in humans, or in animal models, *in vitro* or *in silico* experiments.

**Diagnostic / detection test performance** includes the evaluation of tests such as culture, PCR, rapid antigen tests, whole genome sequencing and others depending on the pathogen being studied. This section would also include studies on the detection of a pathogen in wastewater.

**Genomics and structural characterization** include genomic epidemiology and studies examining mutations of the virus and their impact on structure and fitness.

**Knowledge, attitudes and behaviors** studies. May also include studies of social media sentiment analyses.

**Other epidemiology** includes all studies that document the emergence and spread of the disease in a geographic area, ecological studies that examine spread and risk factors for hot spots, studies on effective public health measures/ infection prevention and control measures/interventions against the disease, studies on animal hosts of the pathogen and zoonotic/zooanthroponosis (if applicable), and analyses of other subcategories when reported.

In the evidence table below, new evidence is highlighted in grey [ADJUST METHOD OF HIGHLIGHTING EVIDENCE AS NEEDED].

# Evidence table

## **Table 1: Evidence on the [INSERT OUTBREAK/EVENT NAME] (n= [INSERT NUMBER OF STUDIES])**

| Category | Key summary outcomes | Overview of the evidence |
| --- | --- | --- |
| Protective immunity (n= number of studies) | | |
| Transmission efficiency | [INSERT EVIDENCE, see example in blue below]  The most recent models of the 2022 outbreak show R_t_ from the end of May – August 2022 has ranged between 1-2.7 across time and countries with the highest number of cases^1,2,3^ which is in line with what previous models^4,5^ suggested, that R_0_>1 can occur for communities with high rates of contacts.   - The Netherlands surveillance data showed a doubling time in June 2022 of 11.2-20.5 days which translated to reproduction numbers of 1.3-1.6 using Lotka-Euler equation.^6^ - A simple SIRD model was used to determine the basic reproduction number (R_0_) which the averaged at 1.28 across countries (UK 1.33, United Arab Emirates 1.08, Nigeria 1.09, Brazil 1.55 and Canada 1.36).^7^ These estimates are lower than other studies.^8,9,10^ | [INSERT STUDY DESIGNS, REFERENCES and LEVEL OF CONFIDENCE, see example below]  Predictive model ^1,4,5,10^ mathematical models^2,3,7,8,9^ and surveillance data analysis.^6^  Low level of evidence |
| Estimates of outbreak size or duration |  |  |
| Secondary attack rate (SAR) |  |  |
| Serial interval |  |  |
| Infectious period |  |  |
| Incubation / latent period |  |  |
| Modes of transmission |  |  |
| Viral kinetics and data on positive samples (positivity, location, virus concentration, and relationship to point in infection) |  |  |
| Asymptomatic, pre-symptomatic transmission |  |  |
| Experimental studies of infectivity |  |  |
| Protective immunity (n= number of studies) | | |
| Clinical characteristics (symptomology and duration) |  |  |
| Virulence / severity epidemiology and/or duration of medical care |  |  |
| Severity risk factors |  |  |
| Mortality epidemiology |  |  |
| Mortality risk factors |  |  |
| Protective immunity (n= number of studies) | | |
| Infection-induced immunity (re-infection after infection) |  |  |
| Pre-exposure vaccination (breakthrough infection / vaccine effectiveness (VE), safety) |  |  |
| Post-exposure vaccination (breakthrough infection, VE, safety) |  |  |
| Experimental studies evaluating vaccine candidates |  |  |
| Therapeutics (n= number of studies) | | |
| Studies of therapeutics (effectiveness/efficacy trials in humans, or preliminary experiments) |  |  |
| Infection, prevention, and control (IPC) (n= number of studies) | | |
| IPC measures in healthcare settings (trials or observational studies) |  |  |
| IPC measures in home/community settings (Trial or observational studies) |  |  |
| IPC general (preliminary experimental data) |  |  |
| Diagnostic / detection test performance (n= number of studies) | | |
| Diagnostic and detection performance |  |  |
| Genomics and structural characterization (n= number of studies) | | |
| Studies of [PATHOGEN] virus mutations |  |  |
| Studies on [PATHOGEN] structural characterization |  |  |
| Knowledge, attitudes, and behaviors (n= number of studies) | | |
| Knowledge, attitudes, and behaviors (KAB) |  |  |
| Other epidemiology (n= number of studies) | | |
| Emergence and spread |  |  |
| Public health measures (PHMs)/ interventions |  |  |
| Adherence to public health measures |  |  |
| Zoonotic (includes transmission to and from animals to humans) |  |  |
| Animal hosts of [PATHOGEN] |  |  |

# Methods

[INSERT METHODS INCLUDING SEARCH DATES, DATABASES SEARCHED, SEARCH TERMS, GREY LITERATURE SEARCHES, ELIGIBILITY CRITERIA, STUDY SELECTION AND METHODS FOR SYNTHESIS].

## **Acknowledgements**

# References
