# Supplementary material for: Methods for conducting a living evidence profile on mpox: An evidence map of the literature
Source: Cochrane Evid Synth Methods. 2024 Feb 22;2(2):e12044. doi: 10.1002/cesm.12044 (PMC11795934; doi:10.1002/cesm.12044)
Supplement: Supplementary file 3 — Supplementary information. [file CESM-2-e12044-s004.docx]

Supplementary Material 3

**Table: Definitions of Foci**

| **Foci** | **Definitions** |
| --- | --- |
| **Transmissibility** | |
| Transmission efficiency | Often predictive or mathematical models that estimate the basic reproduction number R0 or effective reproduction number Rt. The studies evaluate how a pathogen will spread in the population at a specific time / under specified conditions. |
| Estimates of outbreak size or duration | Studies that predict or forecast outbreak size, usually based on mathematical models. E.g., modelling studies that demonstrate cases that were imported could result in a 40-fold increase in cases. |
| Secondary attack rate (SAR) | Research that describes the proportion or percentage of susceptible individuals who become infected after being exposed to an index case. |
| Serial interval | Studies that describe the time from illness onset in the primary case (infected individual) to illness onset in the secondary case. |
| Infectious period | Studies that describe the duration during which a host is capable of directly or indirectly transmitting an infectious agent. |
| Incubation / latent period | Incubation period: Studies that report the interval between exposure to a pathogen and the appearance of the first symptoms of a disease.  Latent period (not often reported): Studies that report the interval between exposure to a pathogen and becoming infectious. |
| Modes of transmission | Research that reports ways in which the virus spreads from an infected host to an uninfected host. This could include airborne transmission, fomite transmission, direct contact, animal to human contact, and studies that describe transmission risk in different settings (e.g., workplace, schools, household). Each mode of transmission could be separated into sub-categories including transmission setting, type or by country were needed. |
| Pathogen kinetics | Studies that describe [PATHOGEN] kinetics which included [PATHOGEN] load at different points during infection, measures of the presence or concentration of viable [PATHOGEN], analysis of different types of samples from the host, the load and survival of the [PATHOGEN] in environmental sampling studies which may report on different sample types (e.g., air, surfaces, hospitals, community settings, etc.). This foci can be separated by sub-categories when needed. |
| Asymptomatic, pre-symptomatic transmission | Citations that report pre-symptomatic (i.e., before symptoms were recognized, but the host is already infectious) or asymptomatic (i.e., no symptoms during infection, but is infectious and can transmit the pathogen) transmission. |
| Experimental studies of infectivity | *In vitro* and *in silico* studies on viral infectivity (i.e., the ability of a virus to enter the host cell and replicated and produce infections viral particles). |
| **Clinical Data** | |
| Clinical characteristics | Includes studies that describe symptomology, frequency of symptoms, and duration of illness. These characteristics can be separated out into sub-categories. These are often descriptive studies initially, followed by cohorts that may be more analytical. |
| Virulence / severity | Articles that report describe disease severity, measure the risk and frequency of severe disease and report on the duration of severe disease typically measured by hospitalizations, ICU, mechanical ventilation and the length of stay at each level of care. |
| Severity risk factors | This include studies describing preexisting attributes of the host (e.g., age, sex or socioeconomic status) or health related factors that are associated with developing severe disease (e.g., being immunocompromised, having comorbidities). This may include being part of a high-risk group (e.g., immunocompromised individuals, pregnant, etc.). The risk factors can be separated out by sub-category. |
| Mortality | Articles that report the disease progression that leads to death or reports the frequency of mortality due to the disease. |
| Mortality risk factors | This include studies describing host attributes or health related risk factors that are associated with a higher risk of mortality from the disease (e.g., comorbidities). |
| **Protective immunity** | |
| Infection-induced immunity (reinfection after infection) | Studies describing infection-induced immunologic protection against reinfection. This can include studies that report the risk of reinfection, those that report neutralizing antibodies after infection to study what constitutes a robust immune response and how long it lasts. |
| Pre-exposure vaccination (breakthrough infection / vaccine effectiveness (VE), safety) | Studies describing impact of pre-exposure vaccination (i.e., vaccination prior to individuals being exposed to the pathogen). This can include studies that assess pre-exposure vaccine effectiveness against breakthrough infections, protection against severe disease (hospitalization) or mortality and safety. Depending on the topic consider creating subgroups for historical (e.g., childhood) vs. recent vaccination, different types of vaccines, changes in dosing etc.  *In vitro* and *in silico* studies reporting immune response and neutralizing antibodies after vaccination are also included in their own sub-category. |
| Post-exposure vaccination (vaccine effectiveness and safety) | Articles describing impact of post-exposure vaccination (i.e., vaccination after individuals have been exposed to the virus). This can include studies that assess post-exposure vaccine effectiveness from infection, protection against severe disease or mortality, and safety.  *In vitro* and *in silico studies* reporting immune response and neutralizing antibodies after vaccination are also included in their own sub-category. |
| Experimental studies evaluating vaccine candidates | Experimental studies such as animal models or *in silico* studies that evaluate vaccine candidates. |
| **Therapeutics** | |
| Studies of therapeutics | Research that evaluates treatments for the disease in an infected host or preliminary experimental studies including *in vitro, in vivo,* or *in silico* studies that evaluated binding of compounds to viral protein complexes. |
| **Infection, prevention, and control (IPC)** | |
| IPC measures in healthcare settings | Studies that reported the evaluation of any infection, prevention and control methods in a healthcare setting. For example, studies that evaluated healthcare worker exposure and use of personal protective equipment in health care settings during care of patients with disease. |
| IPC measures in home/community settings | Articles that evaluated effectiveness of infection, prevention and control methods in a home or community setting. |
| IPC general | Studies that reported preliminary or experimental data on how well an intervention (e.g. disinfectant) could work under controlled conditions. This could include *in vitro* studies evaluating effectiveness of intervention on pathogen inactivation such as hand sanitizers, ultraviolet-C whole room disinfection and heat or *in silico* simulation studies. |
| **Diagnostic / detection test performance** | |
| Diagnostic and detection performance | Articles that describe identification of the pathogen through one or more tests: PCR, sequencing, culture, antibody and/or antigen detection assays, rapid tests, and computer assisted diagnosis. Typically these studies will provide some measure of the tests ability to correctly classify individuals that are diseased or not diseased or classify samples as the pathogen is present or not present. Measures include sensitivity, specificity, positive or negative predictive value, ROC curves, agreement between tests etc. They can be separated by test category and whether it is detection (e.g., wastewater monitoring) or diagnostic (e.g., to diagnose an infection in a human or animal. |
| **Genomics and structural characterization** | |
| Studies of [PATHOGEN] virus mutations | Studies that report phylogenetic analyses and mutations. This can include studies that describe whole genome sequencing to identify large deletions and *in silico* studies that predict changes in the structure of the [PATHOGEN] due to the mutations and its implications. |
| Studies on [PATHOGEN] structural characterization | Research that describes protein-inhibitor interactions such as binding affinity between an inhibitor and a potential antiviral target or studies. |
| **Knowledge, attitudes, and behaviours** | |
| Knowledge, attitudes, and behaviors (KAB) | Studies that evaluated the knowledge, attitudes, and behaviours of the population about the disease, its prevention (including vaccination) and managing the disease. This can include knowledge, intention and motivation to vaccinate, attitudes towards the disease and public health measures, behaviours to reduce risk. Included within this category are internet trend analyses (analysis of tweets and opinions and public interest on the disease via social media data or Google Trend analysis), but these could also be their own sub-category. |
| **Other epidemiology** | |
| Emergence and spread | Articles that report estimates of susceptibility in the population, estimates of spread, growth rate, models that extrapolate wastewater surveillance data and ecological niche modelling. For example, forecasting models that predict trend in cases, models that investigate impact of importation of disease, and studies on first or initial detection of a pathogen (e.g. clinical diagnosis vs. wastewater). |
| Public health measures (PHMs)/ interventions | Studies that evaluate or describe a non-medical intervention to reduce the spread of disease. This can include isolation, quarantine, contact tracing, hand hygiene, masking, and travel restrictions. Many of these will be predictive models, but it is possible to have both experimental and observational evidence that contributes to this foci. |
| Adherence to public health measures | Research that evaluates adherence to recommended or required public health measures. |
| Zoonotic transmission (to and from animals to humans) | Citations that describe animal to human transmission or its reverse, zooanthroponosis, human to animal transmission of a disease. E.g., cases where animals were confirmed to have developed disease days after owner’s symptoms began or studies of how frequently spillover events occur for a zoonotic disease in an endemic area. Depending on the nature of the outbreak or event these foci may need to be split into several sub-foci. |
| Animal hosts of [PATHOGEN] | Experimental or observational studies aimed at establishing what species hosts and/or reservoirs of the pathogen. |
