# Supplementary material for: Methods for conducting a living evidence profile on mpox: An evidence map of the literature
Source: Cochrane Evid Synth Methods. 2024 Feb 22;2(2):e12044. doi: 10.1002/cesm.12044 (PMC11795934; doi:10.1002/cesm.12044)
Supplement: Supplementary file 4 — Supplementary information. [file CESM-2-e12044-s001.docx]

Supplementary Material 4

Certainty of the Evidence.

Information used to establish the certainty of the evidence:

1. Study designs. The evidence pyramid (1) displays the hierarchy of evidence/levels of evidence of study designs. Some study designs are less prone to bias and their estimates are most likely to represent the true value. By design randomized controlled trials control for confounding variables through randomization so have a greater reliability for measuring the effect of an intervention. Below this are other types of studies such as cohort and case series/reports that are at different risk of bias and by their design may not be able to directly measure the outcome of interest. We use study design as an indicator of the strength of the evidence for each foci. The study design is established at the data extraction phase of the LEP and reviewers flag any major concerns with study design and reporting as they extract data.

Confidence in the evidence based on study designs(1):

[Moderate- High] Randomized controlled trials >

[Low – Moderate] controlled trials / prospective cohorts>

[Low] retrospective cohorts / cross-sectional / case control studies >

[Very low – Low] case series / animal models/ challenge trials > predictive models > *in silico / invitro* studies > case reports / other descriptive studies.

1. Number of studies. At the synthesis phase of the LEP we establish how many of each study design contributes to the foci being synthesized. This provides an overview of the quantity of research underpinning the foci and what types of studies have been done to estimate the outcomes in the foci.
2. The volume of literature by study design was considered when five or more decently sized studies measuring the outcome could be considered sufficient to evaluate the consistency in direction and magnitude of the outcome(s) across studies.
   1. First, we consider the direction of effect /association and whether there is agreement across studies. If there are disagreements, outcomes are checked and reasons why there is variation can be examined and discussed.
   2. If there is agreement across studies, then we can also look at the magnitude of the effect/association to see if there is consistency in the magnitude and overlapping confidence intervals. Consistency across studies would improve our confidence that the direction of effect/association is unlikely to change with additional studies and that studies suggest a range of magnitude that is expected variation given the outcome and underpinning studies.
3. The level of certainty in the evidence is a combination of the number of studies for each study design, and consistency in direction and magnitude across the studies. This is reassessed with each cycle.

E.g., An outcome measured in several (>4 studies) low-moderate studies (retrospective cohorts or variable size) to consistently be in the same direction, statistically significant, but with some inconsequential variation in magnitude would be a candidate for upgrading the confidence in the evidence for those foci.

Most foci had studies that offered very low to low certainty of evidence, but as more complicated analytical studies were available and their findings were in agreement with earlier studies, decisions were made to upgrade the certainty in the evidence for some foci one level (e.g., from very low to low or low to moderate. For most outcomes evidence accumulated from descriptive studies to analytical epidemiology studies that were mainly retrospective and prospective cohorts. Studies were upgraded over time to moderate certainty when foci in the LEP had prospective cohorts contributing evidence and there were consistent findings across studies. For foci to be classified as high level of confidence, controlled trial study designs were required.

References:

1. PurdueGlobal. Evidenced Based Practice Pyramid of Resources 2023 [Available from: <https://library.purdueglobal.edu/ebn/ebpyramid>.
